# Interrelationships Among Flow‐Mediated Vasodilation, Nitroglycerine‐Induced Vasodilation, Baseline Brachial Artery Diameter, Hyperemic Shear Stress, and Cardiovascular Risk Factors

**DOI:** 10.1161/JAHA.117.006797

**Published:** 2017-12-29

**Authors:** Tatsuya Maruhashi, Yumiko Iwamoto, Masato Kajikawa, Nozomu Oda, Shinji Kishimoto, Shogo Matsui, Haruki Hashimoto, Yoshiki Aibara, Farina Mohamad Yusoff, Takayuki Hidaka, Yasuki Kihara, Kazuaki Chayama, Kensuke Noma, Ayumu Nakashima, Chikara Goto, Eisuke Hida, Yukihito Higashi

**Affiliations:** ^1^ Department of Cardiovascular Medicine Graduate School of Biomedical and Health Sciences Hiroshima University Hiroshima Japan; ^2^ Department of Cardiovascular Regeneration and Medicine Research Institute for Radiation Biology and Medicine Hiroshima University Hiroshima Japan; ^3^ Department of Medicine and Molecular Science Hiroshima University Graduate School of Biomedical Sciences Hiroshima Japan; ^4^ Division of Regeneration and Medicine Hiroshima University Hospital Hiroshima Japan; ^5^ Hirohsima International University Hiroshima Japan; ^6^ Department of Biostatistics and Data Science Osaka University Graduate School of Medicine Osaka Japan

**Keywords:** brachial artery reactivity, endothelial function, flow‐induced dilation, shear stress, smooth muscle cell, Vascular Biology, Endothelium/Vascular Type/Nitric Oxide, Biomarkers

## Abstract

**Background:**

Flow‐mediated vasodilation (FMD) of the brachial artery has been used for the assessment of endothelial function. Considering the mechanism underlying the vasodilatory response of the brachial artery to reactive hyperemia, hyperemic shear stress (HSS), a stimulus for FMD; nitroglycerine‐induced vasodilation (NID), an index of endothelium‐independent vasodilation; and baseline brachial artery diameter (BAD) are also involved in vasodilatory response. The purpose of this study was to investigate the interrelationships among FMD, HSS, NID, baseline BAD, and cardiovascular risk factors.

**Methods and Results:**

We measured FMD, HSS, NID, and baseline BAD simultaneously in 1033 participants (633 men and 400 women; mean age: 58.6±17.0 years). Framingham risk score was negatively correlated with FMD, HSS, and NID and was positively correlated with baseline BAD. HSS and NID were positively correlated with FMD, and baseline BAD was negatively correlated with FMD. In participants with normal NID, FMD was correlated with HSS, NID, and baseline BAD, all of which were independent variables of FMD in multivariate analysis. In participants with impaired NID, FMD was correlated with NID and baseline BAD, both of which were independent variables of FMD in multivariate analysis, but there was no association between FMD and HSS.

**Conclusions:**

NID and baseline BAD were independent variables of FMD regardless of the status of endothelium‐independent vasodilation, whereas there was a significant association between FMD and HSS in participants with normal NID but not in those with impaired NID. The influence of HSS on FMD seems to be dependent on the status of endothelium‐independent vasodilation.

**Clinical Perspective**

**What Is New?**

- Nitroglycerine‐induced vasodilation and baseline brachial artery diameter were independent variables of flow‐mediated vasodilation in the brachial artery regardless of the status of endothelium‐independent vasodilation.
- The influence of hyperemic shear stress on flow‐mediated vasodilation seems to be dependent on the status of endothelium‐independent vasodilation.
- In participants with normal endothelium‐independent vasodilation, hyperemic shear stress was an independent variable of flow‐mediated vasodilation of the brachial artery, whereas there was no significant association between hyperemic shear stress and flow‐mediated vasodilation in participants with impaired endothelium‐independent vasodilation.

**What Are the Clinical Implications?**

- We should pay attention to endothelium‐independent vasodilation assessed by nitroglycerine‐induced vasodilation for appropriate interpretation of vasodilatory response of the brachial artery to reactive hyperemia.

## Introduction

Endothelial dysfunction is an initial step in the pathogenesis of atherosclerosis and plays an important role in the development and progression of this condition.^1^, ^2^ Moreover, endothelial dysfunction has been shown to be an independent predictor of cardiovascular events.^3^ Therefore, assessment of endothelial function is clinically useful for risk stratification in patients with cardiovascular risk factors.

Flow‐mediated vasodilation (FMD) of the brachial artery has become a widely used technique for assessing endothelial function in humans. This technique measures diameter change of the brachial artery occurring in response to the release of nitric oxide (NO) and other vasoactive substances from the endothelium in response to shear stress during reactive hyperemia.^4^ FMD of the brachial artery indirectly assesses the functional ability of the endothelium to release vasodilating agents, including NO, in response to reactive hyperemia and therefore has been used as an index of endothelial function.^5^ FMD of the brachial artery has been shown to be impaired in patients with cardiovascular risk factors or cardiovascular disease (CVD).^5^

Vasodilatory response of the brachial artery is affected not only by functional status of the endothelium but also by other functional and structural variables of the brachial artery. The mechanism underlying the vasodilatory response of brachial artery to reactive hyperemia is thought to be stimulation of the endothelium by reactive hyperemia‐induced shear stress, leading to the activation of endothelial NO synthase and consequent generation of NO, which diffuses into adjacent vascular smooth muscle cells and interacts with soluble guanylyl cyclase, resulting in relaxation of vascular smooth muscle cells and consequent dilation of the brachial artery. Therefore, in addition to the functional status of the endothelium, hyperemic shear stress (HSS) and endothelium‐independent vasodilation are also involved in vasodilatory response of the brachial artery. Because FMD is calculated as a relative percentage change in the baseline brachial artery diameter (BAD) during reactive hyperemia, baseline BAD is also regarded as an important determinant of FMD.^6^ Previous studies have shown that not only FMD but also HSS; nitroglycerine‐induced vasodilation (NID), an index of endothelium‐independent vasodilation; and baseline BAD are affected in patients with cardiovascular risk factors or CVD.^7^, ^8^, ^9^, ^10^ Therefore, clarifying the interrelationships among FMD, HSS, NID, baseline BAD, and cardiovascular risk factors is important for appropriate interpretation of the vasodilatory response of the brachial artery to reactive hyperemia. Although the associations of FMD with HSS, NID, baseline BAD, and cardiovascular risk factors have been investigated, the relationships among these brachial artery variables and cardiovascular risk factors were not examined simultaneously in any previous studies.^6^, ^7^, ^8^, ^9^

We measured FMD, HSS, NID, and baseline BAD to further determine the interrelationships among these brachial artery variables and cardiovascular risk factors in a large well‐characterized population.

## Methods

The data, analytic methods, and study materials will not be made available to other researchers for purposes of reproducing the results or replicating the procedure.

## Participants

Between July 2007 and February 2016, a total of 2037 participants were recruited for measurement of vascular function from participants who underwent health‐screening examinations or who visited the outpatient clinic at Hiroshima University Hospital. Of the 2037 participants, 1286 underwent measurement of both FMD and NID of the brachial artery. Participants who had received nitrate treatment (n=61) and participants with missing information on Framingham risk score (n=79) and peak blood flow velocity during reactive hyperemia owing to unclear images of the brachial artery after cuff deflation (n=113) were excluded. Finally, 1033 participants (633 men and 400 women; mean age: 58.6±17.0 years) were enrolled in this study. Hypertension was defined as treatment with oral antihypertensive agents or systolic blood pressure ≥140 mm Hg and/or diastolic blood pressure ≥90 mm Hg. Diabetes mellitus was defined according to the American Diabetes Association recommendation or a previous diagnosis of diabetes mellitus.^11^ Dyslipidemia was defined according to the third report of the National Cholesterol Education Program.^12^ Coronary artery disease included angina pectoris with a history of percutaneous coronary intervention and prior myocardial infarction. Cerebrovascular disease included ischemic stroke, hemorrhagic stroke, and transient ischemic attack. Peripheral artery disease was defined as current intermittent claudication with an ankle‐brachial index <0.9 or a history of previous intervention, including angioplasty and bypass grafting. Framingham risk score was calculated by points of risk factors: age, total cholesterol level (or low‐density lipoprotein cholesterol level), high‐density lipoprotein cholesterol level, blood pressure level, diabetes mellitus, and smoking status.^13^ A 10‐year risk of fatal CVD was also estimated using SCORE (Systemic Coronary Risk Evaluation Project) charts by age, systolic blood pressure, total cholesterol, and smoking status.^14^ The ethics committees of our institutions approved the study protocol. Written informed consent for participation in the study was obtained from all participants.

### Study Protocol

We measured vascular responses to reactive hyperemia and sublingually administered nitroglycerine in the brachial artery. The participants fasted the previous night for at least 12 hours. The participants were kept in the supine position in a quiet, dark, air‐conditioned room (constant temperature of 22°C–25°C) throughout the study. A 23‐gauge polyethylene catheter was inserted into the left deep antecubital vein to obtain blood samples. Thirty minutes after maintaining the supine position, baseline BAD and blood flow velocity were measured. Then FMD and hyperemic blood flow velocity during hyperemia were measured. After completion, we next measured NID with confirmation that the BAD had recovered to the baseline value. The observers were blind to the form of examination.

### Measurement of BAD, FMD, Blood Flow Velocity, and NID

Vascular response to reactive hyperemia in the brachial artery was used for assessment of endothelium‐dependent FMD. A high‐resolution linear artery transducer was coupled to computer‐assisted analysis software (UNEXEF18G; UNEX Co) that used an automated edge‐detection system for measurement of BAD. A blood pressure cuff was placed around the forearm. The brachial artery was scanned longitudinally 5 to 10 cm above the elbow. When the clearest B‐mode image of the anterior and posterior intimal interfaces between the lumen and vessel wall was obtained, the transducer was held at the same point throughout the scan by a special probe holder to ensure consistency of the image. Depth and gain were set to optimize the images of the arterial lumen wall interface. When the tracking gate was placed on the intima, the artery diameter was automatically tracked, and the waveform of diameter changes over the cardiac cycle was displayed in real time using the FMD mode of the tracking system. This allowed the ultrasound images to be optimized at the start of the scan and the transducer position to be adjusted immediately for optimal tracking performance throughout the scan. The baseline longitudinal images of the artery were acquired for 10 seconds, and then the blood pressure cuff was inflated to 50 mm Hg above systolic pressure for 5 minutes. Longitudinal images of the artery were recorded continuously until 3 minutes after cuff deflation. Changes in BAD were immediately expressed as percentage change relative to the vessel diameter before cuff inflation. FMD was automatically calculated as the percentage change in peak vessel diameter from the baseline value: (peak diameter−baseline diameter)/baseline diameter. Blood flow velocity was automatically measured at baseline and after cuff deflation for 3 minutes. Blood flow velocity was calculated as time‐averaged flow velocity per cardiac cycle based on cross‐sectional average flow velocity sampled at ≥500 Hz. Blood flow velocity (shown as V) was converted to shear stress according to the following equation: shear stress (dyne/cm^2^)=8×μ×V (cm/s)/BAD (cm). In the equation, μ was blood viscosity assumed to be 0.035 dyne×s/cm^2^. Hyperemic flow velocity was automatically determined as the peak flow velocity obtained after cuff deflation. The hyperemic flow ratio was calculated as the percentage increase in hyperemic flow velocity compared with baseline flow velocity.

The response to nitroglycerine was used for assessment of endothelium‐independent vasodilation. NID was measured as described previously.^9^ Briefly, after acquiring baseline rest images for 30 seconds, a sublingual tablet (75 μg nitroglycerine) was given, and images of the artery were recorded continuously until dilation reached a plateau after administration of nitroglycerine. Participants in whom the sublingually administered nitroglycerine tablet was not dissolved during the measurement were excluded from this study. NID was automatically calculated as a percentage change in peak vessel diameter from the baseline value: (peak diameter−baseline diameter)/baseline diameter.

### Statistical Analyses

Results are presented as mean±SD. All reported probability values were 2‐sided, and a probability value of <0.05 was considered statistically significant. Categorical variables were compared by means of the χ^2^ test. Univariate linear regression analyses were performed to assess the relationships among the variables. Multivariate regression analyses using forward stepwise selection were performed to identify independent variables associated with FMD, HSS, NID, and baseline BAD from the following covariates with *P*<0.05 for inclusion: body mass index, systolic blood pressure, heart rate, total cholesterol, triglycerides, high‐density lipoprotein cholesterol, low‐density lipoprotein cholesterol, glucose, diabetes mellitus, current smoking, antihypertensive drug treatment, statin treatment, CVD, and other brachial variables, with age and sex forced into the model. Because age has been shown to be strongly associated with brachial variables and to be related to other cardiovascular risk factors,^6^, ^7^, ^9^, ^15^ we had planned to test the statistical interactions between age and other variables. However, we found no evidence of a qualitative interaction between age and other variables analytically and graphically. Therefore, no interaction terms were included in the final regression models. There were significant linear relationships between brachial variables and all covariates remaining in the final regression models. Because reference values of FMD, HSS, NID, and baseline BAD have not been fully determined, we did not perform categorical analyses. We examined variance inflation factors for the assessment of multicollinearity. In all multivariate regression analyses, values of the variance inflation factors of all variables were small (<2), indicating that there were no interactions between variables. Adjusted *r* ^2^ is a measure of the proportion of variation in a dependent variable explained by potential covariates, providing an estimate of the strength of the relationship between the linear model and covariates. The data were processed using the software package Stata version 9 (StataCorp).

## Results

### Baseline Clinical Characteristics

The baseline clinical characteristics of the participants are summarized in Table 1. Of the 1033 participants, 633 (61.3%) were men, 723 (70.1%) had hypertension, 565 (54.7%) had dyslipidemia, 317 (30.7%) had diabetes mellitus, 214 (20.7%) were current smokers, and 237 (23.2%) had CVD. Mean values were 3.9±2.8% for FMD, 12.2±5.7% for NID, 4.10±0.65 mm for baseline BAD, 6.4±3.8 dyne/cm^2^ for baseline shear stress, and 24.0±15.4 dyne/cm^2^ for HSS.

**Table 1:** Clinical Characteristics of the Participants (N=1033)

Variables	Results
Age, y	58.6±17.0
Men, n (%)	633 (61.3)
Body mass index, kg/m^2^	23.4±3.9
Systolic blood pressure, mm Hg	131.9±18.9
Diastolic blood pressure, mm Hg	78.1±12.5
Heart rate, beats/min	69.9±12.1
Total cholesterol, mg/dL	191.4±36.8
Triglycerides, mg/dL	139.3±95.5
HDL cholesterol, mg/dL	59.2±16.7
LDL cholesterol, mg/dL	111.6±32.6
Glucose, mg/dL	113.6±40.1
Hypertension, n (%)	723 (70.1)
Dyslipidemia, n (%)	565 (54.7)
Diabetes mellitus, n (%)	317 (30.7)
Current smokers, n (%)	214 (20.7)
Antihypertensive drug treatment, n (%)	598 (58.4)
CVD, n (%)	237 (23.2)
Coronary heart disease, n (%)	126 (12.3)
Cerebrovascular disease, n (%)	67 (6.5)
Peripheral artery disease, n (%)	95 (9.4)
Framingham risk score, %	10.5±8.8
SCORE risk, %	2.2±2.3
Flow‐mediated vasodilation, %	3.9±2.8
NID, %	12.2±5.7
Baseline BAD, mm	4.10±0.65
Baseline	
Flow velocity, cm/s	9.3±5.6
Shear stress, dyne/cm^2^	6.4±3.8
Hyperemia	
Flow velocity, cm/s	34.8±22.3
Shear stress, dyne/cm^2^	24.0±15.4
Hyperemic flow ratio	4.0±2.0

BAD indicates brachial artery diameter; CVD, cardiovascular disease; HDL, high‐density lipoprotein; LDL, low‐density lipoprotein; NID, nitroglycerine‐induced vasodilation; SCORE, Systemic Coronary Risk Evaluation Project.

### Relationships Among FMD, HSS, NID, Baseline BAD, and Cardiovascular Risk Factors

Relationships between brachial artery variables and cardiovascular risk factors are presented in Table 2. Framingham risk score was negatively correlated with FMD (β=−0.708, *r*=−0.23, *P*<0.001), HSS (β=−0.041, *r*=−0.07, *P*=0.020), and NID (β=−0.275, *r*=−0.18, *P*<0.001) and was positively correlated with baseline BAD (β=3.094, *r*=0.23, *P*<0.001). SCORE risk at 10 years was negatively correlated with FMD (β=−0.189, *r*=−0.23, *P*<0.001) and NID (β=−0.072, *r*=−0.18, *P*<0.001) and was positively correlated with baseline BAD (β=0.733, *r*=0.20, *P*<0.001), although there was no significant correlation between SCORE risk and HSS (β=−0.001, *r*=−0.01, *P*=0.772). These findings suggest that FMD, HSS, and NID decrease and baseline BAD increases in relation to cumulative cardiovascular risk factors.

**Table 2:** Univariate Analysis of the Relationships Between Brachial Artery Variables and Risk Factors

Variables	FMD			HSS			NID			Baseline BAD		
	β	*r*	*P* Value	β	*r*	*P* Value	β	*r*	*P* Value	β	*r*	*P* Value
Age, y	−2.171	−0.36	<0.001	−0.139	−0.13	<0.001	−0.935	−0.32	<0.001	3.181	0.12	<0.001
Body mass index, kg/m^2^	−0.094	−0.07	0.028	−0.048	−0.19	<0.001	−0.048	−0.07	0.024	1.693	0.28	<0.001
Systolic blood pressure, mm Hg	−0.794	−0.12	<0.001	−0.052	−0.04	0.174	−0.479	−0.15	<0.001	2.868	0.10	0.002
Diastolic blood pressure, mm Hg	−0.448	−0.10	0.001	−0.055	−0.07	0.030	−0.024	−0.01	0.721	3.174	0.16	<0.001
beats/min	0.018	−0.004	0.891	−0.040	−0.05	0.102	−0.092	−0.04	0.164	−0.533	−0.03	0.362
Total cholesterol, mg/dL	−0.916	−0.07	0.027	−0.229	−0.10	0.003	−0.107	−0.02	0.603	−1.853	−0.03	0.311
Triglycerides, mg/dL	−2.962	−0.09	0.005	−0.322	−0.05	0.100	0.534	0.03	0.304	11.160	0.08	0.016
HDL cholesterol, mg/dL	0.077	0.01	0.673	0.047	0.04	0.161	−0.022	−0.01	0.809	−4.516	−0.18	<0.001
LDL cholesterol, mg/dL	−0.634	−0.06	0.075	−0.179	−0.08	0.007	−0.220	−0.04	0.213	0.976	0.02	0.535
Glucose, mg/dL	−2.411	−0.17	<0.001	−0.059	−0.02	0.483	−0.299	−0.04	0.193	0.851	0.01	0.676
Framingham risk score, %	−0.708	−0.23	<0.001	−0.041	−0.07	0.020	−0.275	−0.18	<0.001	3.094	0.23	<0.001
SCORE risk, %	−0.189	−0.23	<0.001	−0.001	−0.01	0.772	−0.072	−0.18	<0.001	0.733	0.20	<0.001

BAD indicates brachial artery diameter; FMD, flow‐mediated vasodilation; HDL, high‐density lipoprotein; HSS, hyperemic shear stress; LDL, low‐density lipoprotein; NID, nitroglycerine‐induced vasodilation; SCORE, Systemic Coronary Risk Evaluation Project.

### Relationships Among FMD, HSS, NID, and Baseline BAD

HSS (β=0.519, *r*=0.10, *P*=0.002) and NID (β=0.759, *r*=0.38, *P*<0.001) were positively correlated with FMD, and baseline BAD (β=−0.090, *r*=−0.40, *P*<0.001) was negatively correlated with FMD, indicating that FMD decreases in relation to decrease in HSS and NID and in relation to increase in baseline BAD (Table 3). Baseline BAD was also negatively correlated with HSS (β=−2.992, *r*=−0.13, *P*<0.001) and NID (β=−3.295, *r*=−0.40, *P*<0.001; Table 3). Baseline BAD was an independent variable of HSS (β=−0.114, *P*=0.002) and NID (β=−0.490, *P*<0.001) in multivariate analyses (Tables 4 and 5), indicating that NID and HSS decrease in relation to increase in baseline BAD. In multiple linear regression analysis of the relationships between FMD and variables, when no consideration was given to brachial artery variables, age, sex, triglycerides, glucose, antihypertensive drug treatment, and statin treatment were independent variables of FMD (Table 6). When HSS was entered into the model, HSS was not associated with FMD (β=0.041, *P*=0.170) without an increase in adjusted *r* ^2^ of the model (Table 6). When NID was considered as an additional covariate, NID was an independent variable of FMD (β=0.286, *P*<0.001) with an increase in adjusted *r* ^2^ from 0.18 to 0.25 (Table 6). Moreover, when baseline BAD was additionally entered into the model, baseline BAD was also an independent variable of FMD (β=−0.305, *P*<0.001) with a further increase in adjusted *r* ^2^ from 0.25 to 0.31, and NID remained significant (β=0.170, *P*<0.001; Table 6).

**Table 3:** Univariate Analysis of the Relationships Among FMD, HSS, NID, and BAD

Variables	HSS		NID		Baseline BAD	
	β	*r*	β	*r*	β	*r*
FMD	0.519	0.10^a^	0.759	0.38^b^	−0.090	−0.40^b^
Baseline BAD	−2.992	−0.13^b^	−3.295	−0.40^b^	···	···

BAD indicates brachial artery diameter; FMD, flow‐mediated vasodilation; HSS, hyperemic shear stress; NID, nitroglycerine‐induced vasodilation.

a *P*<0.01.

b *P*<0.001.

**Table 4:** Multiple Linear Regression Analysis of the Relationships Among HSS and Variables

Variables	HSS			
	β	VIF	SE	*P* Value
Age, y	−0.087	1.10	0.029	0.072
Men	−0.123	1.36	0.567	<0.001
Body mass index, kg/m^2^	−0.163	1.11	0.127	<0.001
Total cholesterol, mg/dL	−0.064	1.05	0.013	0.044
Baseline BAD, mm	−0.114	1.37	0.859	0.002

The adjusted *r* ^2^ was 0.06. BAD indicates brachial artery diameter; HSS, hyperemic shear stress; VIF, variance inflation factor.

**Table 5:** Multiple Linear Regression Analysis of the Relationships Among NID and Variables

Variables	NID			
	β	VIF	SE	*P* Value
Age, y	−0.123	1.35	0.010	<0.001
Men	0.274	1.35	0.179	<0.001
Body mass index, kg/m^2^	0.078	1.22	0.042	0.007
Systolic blood pressure, mm Hg	−0.097	1.08	0.008	<0.001
Antihypertensive drug treatment	−0.128	1.26	0.172	<0.001
CVD	−0.086	1.10	0.187	0.002
Baseline BAD, mm	−0.490	1.38	0.276	<0.001

The adjusted *r* ^2^ was 0.31. BAD indicates brachial artery diameter; CVD, cardiovascular disease; NID, nitroglycerine‐induced vasodilation; VIF, variance inflation factor.

**Table 6:** Multiple Linear Regression Analysis of the Relationships Among FMD and Variables

Variables	Model 1				Model 2				Model 3				Model 4			
	β	VIF	SE	*P* Value	β	VIF	SE	*P* Value	β	VIF	SE	*P* Value	β	VIF	SE	*P* Value
Age, y	−0.376	1.41	0.006	<0.001	−0.372	1.42	0.006	<0.001	−0.290	1.51	0.006	<0.001	−0.260	1.53	0.006	<0.001
Men	0.069	1.06	0.090	0.025	0.071	1.06	0.090	0.021	0.084	1.06	0.086	0.004	0.060	1.42	0.096	0.066
Triglycerides, mg/dL	−0.109	1.06	0.001	<0.001	−0.107	1.06	0.001	<0.001	−0.110	1.06	0.001	<0.001	−0.094	1.07	0.001	<0.001
Glucose, mg/dL	−0.075	1.10	0.002	0.016	−0.076	1.10	0.002	0.015	−0.083	1.10	0.002	0.005	−0.089	1.10	0.002	0.002
Antihypertensive drug treatment	−0.120	1.17	0.187	<0.001	−0.117	1.18	0.094	<0.001	−0.077	1.20	0.090	0.011	−0.068	1.20	0.087	0.024
Statin treatment	−0.106	1.23	0.201	0.001	−0.106	1.23	0.201	0.001	−0.010	1.23	0.193	0.013	−0.078	1.23	0.185	0.010
HSS, dyne/cm^2^	···	···	···	···	0.041	1.03	0.006	0.170	0.029	1.03	0.005	0.317	−0.004	1.05	0.006	0.895
NID, %	···	···	···	···	···	···	···	···	0.286	1.15	0.015	<0.001	0.170	1.38	0.016	<0.001
Baseline BAD, mm	···	···	···	···	···	···	···	···	···	···	···	···	−0.305	1.59	0.154	<0.001

The adjusted *r* ^2^ was 0.18 for model 1, 0.18 for model 2, 0.25 for model 3, and 0.31 for model 4. BAD indicates brachial artery diameter; FMD, flow‐mediated vasodilation; HSS, hyperemic shear stress; NID, nitroglycerine‐induced vasodilation; VIF, variance inflation factor.

To further define the interrelationships among brachial artery variables, participants were divided into 2 groups based on the median NID: those with normal NID (≥11.9%) and those with impaired NID (<11.9%). In participants with normal NID, FMD was positively correlated with HSS (*r*=0.13, *P*=0.004; Figure A) and NID (*r*=0.22, *P*<0.001) and negatively correlated with baseline BAD (*r*=−0.30, *P*<0.001), all of which were independent variables of FMD in multivariate analysis (β=0.080, *P*=0.048 for HSS; β=0.125, *P*=0.003 for NID; β=−0.328, *P*<0.001 for BAD; Table 7). In contrast, in participants with impaired NID, FMD was positively correlated with NID (*r*=0.22, *P*<0.001) and negatively correlated with baseline BAD (*r*=−0.38, *P*<0.001), but there was no significant relationship between FMD and HSS (*r*=0.0005, *P*=0.991) (Figure B). NID (β=0.084, *P*=0.044) and baseline BAD (β=−0.348, *P*<0.001) were independent variables of FMD in multivariate analysis in participants with impaired NID (Table 7).

**Figure 1:**
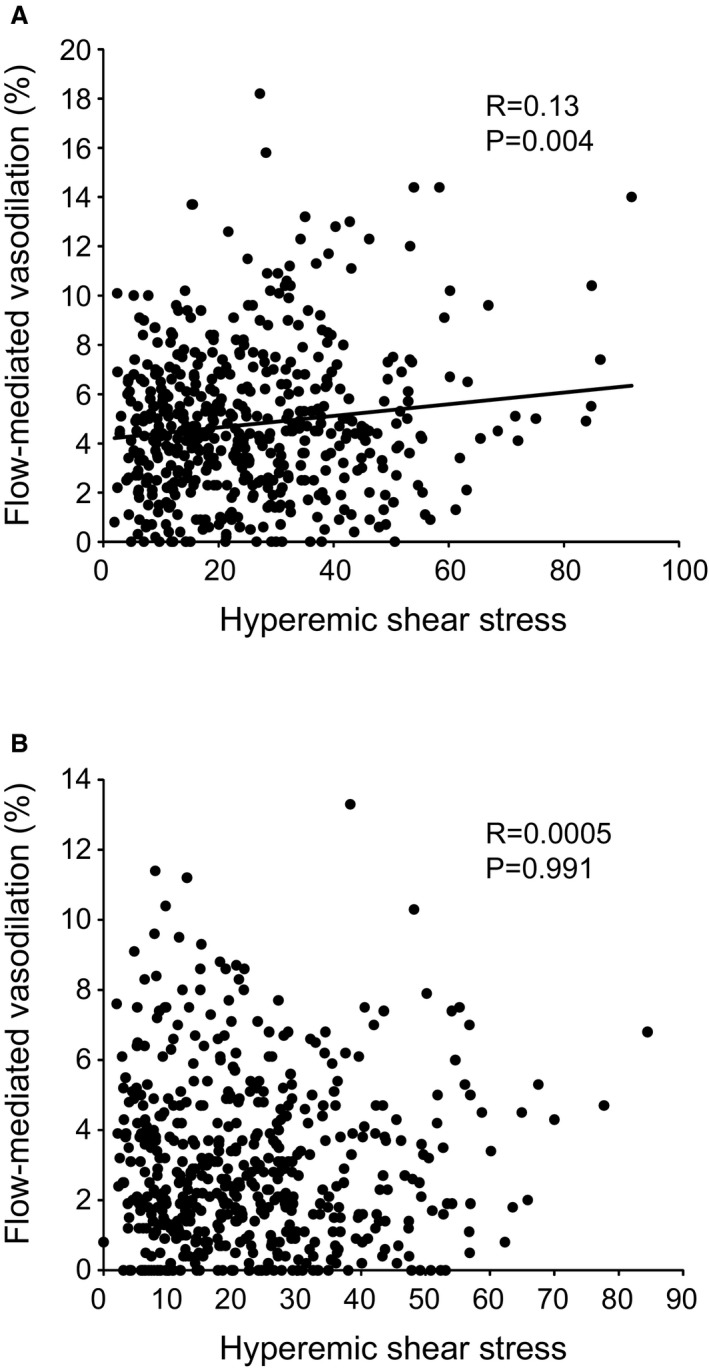
Scatter plots show the relationship between flow‐mediated vasodilation and hyperemic shear stress in participants with normal (A) and impaired (B) nitroglycerine‐induced vasodilation.

**Table 7:** Multiple Linear Regression Analysis of the Relationships Among FMD and Variables Based on the Median of NID

Variables	Normal NID				Impaired NID			
	β	VIF	SE	*P* Value	β	VIF	SE	*P* Value
Age, y	−0.277	1.17	0.007	<0.001	−0.229	1.33	0.007	<0.001
Men	−0.081	1.54	0.305	0.096	−0.048	1.34	0.219	0.293
Body mass index, kg/m^2^	0.129	1.29	0.033	0.004	···	···	···	···
Triglycerides, mg/dL	−0.141	1.17	0.001	0.001	···	···	···	···
Glucose, mg/dL	−0.132	1.16	0.003	0.002	···	···	···	···
Antihypertensive drug treatment	···	···	···	···	−0.134	1.10	0.106	0.001
Statin treatment	···	···	···	···	−0.156	1.21	0.214	<0.001
HSS, dyne/cm^2^	0.080	1.07	0.007	0.048	···	···	···	···
NID, %	0.125	1.14	0.031	0.003	0.084	1.14	0.034	0.044
Baseline BAD, mm	−0.328	1.73	0.271	<0.001	−0.348	1.36	0.164	<0.001

The adjusted *r* ^2^ of the model for participants with normal NID was 0.27. The adjusted *r* ^2^ of the model for participants with impaired NID was 0.23. BAD indicates brachial artery diameter; FMD, flow‐mediated vasodilation; HSS, hyperemic shear stress; NID, nitroglycerine‐induced vasodilation; VIF, variance inflation factor.

## Discussion

In this study, we demonstrated that FMD, HSS, and NID decreased and that baseline BAD increased in relation to increase in cardiovascular risk. Lower HSS, smaller NID, and larger baseline BAD were related to smaller FMD. Regardless of impairment of NID, NID and baseline BAD were independent predictors of FMD. In contrast, HSS was associated with FMD in participants with normal NID but not in those with impaired NID. To our knowledge, this report is the first showing the interrelationships among FMD, HSS, NID, baseline BAD, and cardiovascular risk factors simultaneously and showing the difference in the association of HSS and FMD depending on the status of NID.

Although the relationships between brachial artery variables and cardiovascular risk factors have been investigated in a few studies with large numbers of participants, NID was not measured, and the interrelationships among FMD, HSS, NID, baseline BAD, and cardiovascular risk factors were not examined simultaneously in any previous studies.^7^, ^8^ In this study, measurement of NID was performed in a large number of participants, and this allowed us to investigate the interrelationships between brachial artery variables and cardiovascular risk factors simultaneously. As for the associations of cardiovascular risk factors with brachial artery variables, previous studies demonstrated that HSS and NID decrease and that BAD increases in relation to an increase in cardiovascular risk.^7^, ^8^, ^9^, ^16^ Decreased reactive hyperemic flow velocity and HSS might reflect microvascular dysfunction because reactive hyperemia is strongly dependent on maximal forearm resistance and has been shown to be, at least in part, an NO‐dependent process.^17^, ^18^ Although, to our knowledge, no clinical investigation has shown that administration of an antioxidant such as vitamin C improves vasodilatory response to nitroglycerine, the involvement of oxidative stress in impaired vascular response to nitroglycerine has been indicated in in vitro and in vivo studies. Impairment of NID might reflect vascular smooth muscle cell dysfunction, including inhibited activity of soluble guanylyl cyclase and consequent activation of cGMP‐dependent kinase by oxidative stress, and vascular structural alterations, including increased connective tissue matrix in thickened intima–media layers and consequent limitation of relaxation as a result of atherosclerosis.^19^, ^20^ An increase in oxidative stress may also be associated with attenuated biotransformation of nitroglycerine in patients with cardiovascular risk. Sydow et al demonstrated that oxidative stress attenuated biotransformation of nitroglycerine by inhibition of mitochondrial aldehyde dehydrogenase activity involved in the process of nitroglycerine biotransformation, raising the possibility that attenuation of nitroglycerine biotransformation by oxidative stress may contribute to the impairment of NID in patients with cardiovascular risk.^21^ A previous study showed that the brachial artery tends to be larger in patients with cardiovascular risk factors and CVD.^22^ Enlargement of the brachial artery may occur in response to increasing blood flow to maintain shear stress in an appropriate range, which is important for maintenance of the properly functioning endothelium, as a consequence of adaptive remodeling in patients with cardiovascular risk factors and CVD, including obesity, hypertension, dyslipidemia, coronary artery disease, and peripheral artery disease; in contrast, older age is associated with a larger brachial artery regardless of lower shear stress in the brachial artery, suggesting maladaptive remodeling.^22^ Consistent with these previous observations, our results showed that HSS and NID decreased and that baseline BAD increased in relation to an increase in Framingham risk score, a risk calculator and an index of cumulative cardiovascular risk commonly used for assessing the probability of heart attack or death from heart disease within 10 years; this finding indicates that higher cardiovascular risk is associated with lower HSS, smaller NID, and larger baseline BAD.

As for the interrelationships among the brachial artery variables, consistent with results of previous studies, HSS and NID were positively correlated with FMD and baseline BAD was negatively correlated with FMD in this study.^6^, ^7^, ^8^, ^9^ Because HSS is a stimulus for vasodilatory response, lower HSS is likely to be associated with a smaller vasodilatory response, resulting in attenuation of FMD. Because FMD caused by reactive hyperemia occurs as a result of vascular smooth muscle relaxation, impaired NID, as an index of endothelium‐independent vasodilation reflecting, at least in part, the ability of vascular smooth muscle to relax in response to exogenous NO, may result in impaired vasodilatory response to reactive hyperemia regardless of the status of endothelial function. Because FMD is calculated as a relative percentage change in baseline BAD during reactive hyperemia, larger baseline BAD is associated with smaller FMD. In addition, in the present study, baseline BAD was negatively correlated with HSS and NID and was an independent predictor of both HSS and NID. HSS seems to be lower in larger brachial arteries because of the dependence of postischemic systolic flow on radius squared.^23^ NID, which is calculated as a relative percentage change in baseline BAD in response to administered nitroglycerine, is smaller in a larger baseline brachial artery.^9^ Our results suggest that larger baseline BAD is associated with smaller FMD not only directly but also indirectly through lowered HSS and smaller NID.

In the present study, NID and baseline BAD were independent variables of FMD, but HSS was not in all participants. Mitchell et al reported that HSS was an independent predictor of FMD in a large community‐based cohort in the Framingham Heart Study.^7^ Philpott et al also reported that HSS was an independent predictor of FMD in a relatively young and healthy population in the FATE (Firefighters and Their Endothelium) study.^8^ Although we have no direct explanation for the discrepancy between the results of those previous studies and the results of the present study, the difference in the relationship between FMD and HSS might be related to the difference in the status of endothelium‐independent vasodilation owing to participant selection. We previously reported that NID of the brachial artery was impaired in patients with multiple cardiovascular risk factors or established CVD.^9^ In these patients with advanced atherosclerosis, no matter how high HSS is, vasodilatory response of the brachial artery might be impaired owing to the impaired endothelium‐independent vasodilation involved directly in the vasodilatory response to reactive hyperemia. Participants in the present study had higher prevalence of hypertension, diabetes mellitus, and established CVD and included a larger percentage of current smokers than those in the previous studies. Consequently, impaired endothelium‐independent vasodilation might have made the relationship between FMD and HSS weak in the present study, whereas there was a significant association between FMD and HSS in the previous studies, in which the participants were recruited from a general population or were young healthy adults in whom endothelium‐independent vasodilation was assumed to be normally maintained. Indeed, when participants were divided into 2 groups based on median NID in the present study, there was a significant positive association between FMD and HSS in participants with normal NID, whereas there was no significant association between FMD and HSS in those with impaired NID, indicating that the interrelation between FMD and HSS is altered depending on the status of endothelium‐independent vasodilation. NID and baseline BAD were independent predictors of FMD regardless of the status of endothelium‐independent vasodilation.

In conclusion, higher cardiovascular risk is associated with lower HSS, smaller NID, and larger baseline BAD, all of which are related to smaller FMD of the brachial artery. Cardiovascular risk factors are not only directly but also indirectly associated with impairment of FMD through lower HSS, smaller NID, and larger BAD. NID and baseline BAD were independent variables of FMD regardless of the status of endothelium‐independent vasodilation. In contrast, the influence of HSS on FMD seems to be dependent on the status of endothelium‐independent vasodilation. In participants with normal endothelium‐independent vasodilation, HSS was an independent variable of FMD, whereas there was no significant association between HSS and FMD in participants with impaired NID. We should pay attention to endothelium‐independent vasodilation assessed by NID for appropriate interpretation of vasodilatory response of the brachial artery to reactive hyperemia.

## Sources of Funding

This work was supported by a Grant‐in‐Aid for Scientific Research from the Ministry of Education, Science and Culture of Japan (18590815 and 21590898 to Higashi).

## Disclosures

None.
